# Preventing Uterine Cervix Cancer: The Clinical Meaning of Atypical Glandular Cells

**DOI:** 10.1055/s-0042-1742318

**Published:** 2022-02-09

**Authors:** Gutemberg Almeida, Jorge Eduardo Sainz, Renata Fonseca, Neil Chaves, Katia Silveira Silva, Julio Nunes, Yara Furtado

**Affiliations:** 1 Universidade Federal do Rio de Janeiro, Rio de Janeiro, RJ, Brazil; 2 Instituto Fernandes Figueira, Rio de Janeiro, RJ, Brazil; 3 Department of Psychiatry and Behavior Sciences, Stanford University, Stanford, CA, United States; 4 Universidade Federal do Estado do Rio de Janeiro, Rio de Janeiro, RJ, Brazil

**Keywords:** AGC, atypical glandular cells, glandular cervical neoplasia, Bethesda system, cervical cancer screening, cytology, AGC, células glandulares atípicas, neoplasia glandular cervical, sistema Bethesda, rastreio do câncer do colo uterino, citologia

## Abstract

**Objective** To determine the prevalence of the atypical glandular cells (AGCs) cytology and to analyze its clinical significance in different age ranges.

**Methods** Retrospective observational study using computerized data from the Brazilian National Cancer Institute, including women screened between January 2002 and December 2008. The women included were those with an AGC result who were properly followed-up with colposcopy and a second cytology.

**Results** A total of 132,147 cytopathological exams were performed during the study period. Five-hundred and thirty-three (0.4%) women with AGC cytology were identified and, of these, 69.41% (370/533) were properly referred for colposcopy and a new cytology. Most of the women (79.2%) with a 1 ^st^ or 2 ^nd^ AGC cytology were between the ages of 25 and 54 years. The 2 ^nd^ cytology demonstrated 67.6% (250/370) of normality, 24.5% (91/370) of squamous atypia, and 6.2% (23/370) of AGC, 0.8% (3/370) adenocarcinoma in situ and 0.8% (3/370) adenocarcinoma invasor. On biopsy of the women with a second AGC cytology, 43.4% (10/23) had normal histology, 43.4% (10/23) had squamous lesions, 8.7% (2/23) had invasive adenocarcinoma, and 1.2% (1/23) had an inconclusive report. All of the women with high-grade squamous intraepithelial lesion (HSIL) or invasive adenocarcinoma (respectively 5 and 2 patients), after a 2 ^nd^ AGC cytology were 25 years old or older.

**Conclusion** The prevalence of the AGC cytology was low in the studied population. Most of the AGC cytology cases occurred in adult women between the ages of 25 and 54. Although most of the patients had normal histology after follow-up, several of them presented with squamous intraepithelial lesions or invasive adenocarcinoma.

## Resumo

**Objetivo** Determinar a prevalência de citologia com laudo de células glandulares atípicas (AGCs, na sigla em inglês) e analisar a significância clínica nas diferentes faixas etárias

**Métodos** Estudo observacional retrospectivo, usando os dados arquivados no sistema do Instituto Nacional de Câncer no Brasil, que incluiu mulheres rastreadas entre janeiro de 2002 a dezembro de 2008. As mulheres incluídas tinham citologia com resultado de AGCs, que foram acompanhadas com colposcopia e nova citologia

**Resultados** Um total de132,147 exames citopatológicos foram incluídos durante o período de estudo. Quinhentas e trinta e três mulheres com citologia de AGC foram identificadas e destas, 69.41% (370) foram encaminhadas para colposcopia e nova citologia. A prevalência de citologia de AGC na população estudada foi 0.4%. A maioria das mulheres (79.22%) com resultado citológico de AGC tinham idade entre 25 e 54 anos. A segunda citologia demonstrou 67.56% (250/370) de normalidade, 24.5% (91/370) de atipias escamosas, e 6.2% (23/370) de AGC. Na biopsia das mulheres com a 2ª citologia de AGC, 43.4% (10/23) tinham histologia normal, 43.4% (10/23) tinha lesões escamosas, 8.7% (2/23) tinha adenocarcinoma invasor e 1.2% (1/23) tinha laudo inconclusivo. Todas as mulheres com lesões intraepiteliais escamosas de alto grau (HSIL, na sigla em inglês) ou adenocarcinoma invasor (respectivamente 5 e 2 pacientes), após a 2ª citologia com AGC, tinham 25 anos de idade ou mais.

**Conclusão** A prevalência de citologia com AGC foi baixa na população estudada. Muitos casos de citologia com AGC apareceram em mulheres adultas, entre 25 e 54 anos de idade. Embora a maioria das pacientes tiveram histologia normal após seguimento, várias apresentaram lesões intraepiteliais escamosas ou glandulares invasoras.

## Introduction

Several cervical-vaginal cytological classification systems have been suggested since the Papanicolaou and Traut, 1 but, currently, the most used one in the world is the Bethesda system. 2 3 4 Developed in December 1988, it suggested including lesions related to the human papillomavirus (HPV) and grade I cervical intraepithelial neoplasia (CIN I) in the same category, called low-grade squamous intraepithelial lesions (LSILs). Also, CIN II/III should be categorized as high-grade squamous intraepithelial lesions (HSILs). 3

Bethesda also introduced undetermined categories: atypical squamous cells of undetermined significance (ASCUS) and atypical glandular cells of undetermined significance (AGUS). 3 These were lesions with microscopic reactive changes unusual for benign processes, but not notable enough for the accurate diagnosis of adenocarcinoma. 3

In 2001, in a revision, the Bethesda system renamed AGUS as atypical glandular cells (AGCs) cytology. 4 It recommended characterizing AGC according to its anatomical origin: endocervical, endometrial, or of unspecified origin (AGC not otherwise specified—NOS). A new subcategory for AGC suspicious for neoplasia (AGC *favor* neoplasia) was included. 4 All these changes were maintained in the last review of the Bethesda system, in 2015. 5

In the literature, AGC is present in less than 1% of cytological samples, with an incidence varying from 0.1 to 2.1%. 6 7 8 9 10 11 In the United States, its prevalence was 0.4% in 2003. 10 11 In Brazil, it corresponded to 4.6% of the altered cytologic exams performed in 2009. 12

Despite the low prevalence of AGC, this diagnosis holds high importance due to its high frequency of association with neoplastic changes (e.g., squamous intraepithelial neoplasia, adenocarcinoma in situ, invasive adenocarcinomas of the cervix and endometrium, and, more rarely, extrauterine neoplasms). Other benign findings, such as vaginal adenosis, endometrial and endocervical polyps, inflammatory conditions, and reactive changes, may also be related to this cytological change. 13

A systematic review by Marques et al. *(* 2011) 14 assessed the association between the diagnosis of AGC and the occurrence of benign and/or premalignant or invasive lesions of the cervix. They observed a significant relation between AGC and benign disease. Nevertheless, the frequency of invasive squamous carcinoma (in patients previously diagnosed with AGC) ranged from 0.89 to 4.44%, and that of invasive adenocarcinoma ranged from 1.4 to 18%. 14

International protocols do not yet establish a consensus regarding the referral of patients with a cytopathological diagnosis of AGC. In the Brazilian Guidelines for Cervical Cancer Screening (2016), used in our population, patients with AGC should be immediately referred to a second cytology test (including material from the endocervical canal) and colposcopy. 15

If the endocervical cytology result is adenocarcinoma in situ (AIS) or HSIL, prompt excisional treatment should follow. During colposcopy, if changes of any nature appear, a biopsy is necessary for therapeutic planning. 16 Physicians should employ an excision technique that produces an intact specimen for adequate evaluation of its margins. 17

In women, those with AGC, older than 35 years or AGC with abnormal uterine or AGC of endometrial origin, endometrial evaluation must be considered (with ultrasound and/or biopsy). 15

The present study aimed to determine the prevalence and frequency of the AGC cytology and to evaluate its clinical significance in various age ranges.

## Methods

Retrospective observational study, using computerized data from the Integrated System of Technology and Cytopathology (SITEC, in the Portuguese acronym), Division of Pathology, from the Brazilian National Cancer Institute (INCA, in the Portuguese acronym). The SITEC is responsible for processing cytological examinations performed in a major part of Rio de Janeiro, Brazil. Therefore, it produces a comprehensive database of medical records. Files dated from January 2002 to December 2008 were evaluated in search of women diagnosed with AGC cytology. The women included were those with an AGC result and then referred to colposcopy and a second cytologic study (including material from the endocervical canal), as recommended by the Brazilian Guidelines for Cervical Cancer Screening (2016). 15 All of the follow-up procedures (colposcopy, second cytology and, possibly, biopsy) were performed at the same reference facility (Posto de Assistência Médica Manoel Guilherme da Silveira Filho, in Rio de Janeiro). When a colposcopy evidenced abnormal findings, a biopsy was the next step in management. Reports were standardized according to the nomenclature guidelines established by the Brazilian Ministry of Health and the Brazilian Society of Cytopathology. 6 Statistically, the prevalence of the AGC cytology was determined, the histological frequency of atypical glandular and squamous cervical lesions was calculated, and the frequency of disagreement between the cytological and histological exams was ascertained. The age ranges were organized (14–24, 25–34, 35–44, 45–54, 55–64, > 64). This project was approved by Ethics Committee of Maternidade Escola da Univerisdade Federal do Rio de Janeiro (Ethics Committee Regulation Number 10/2011).

## Results

**Fig. 1:**
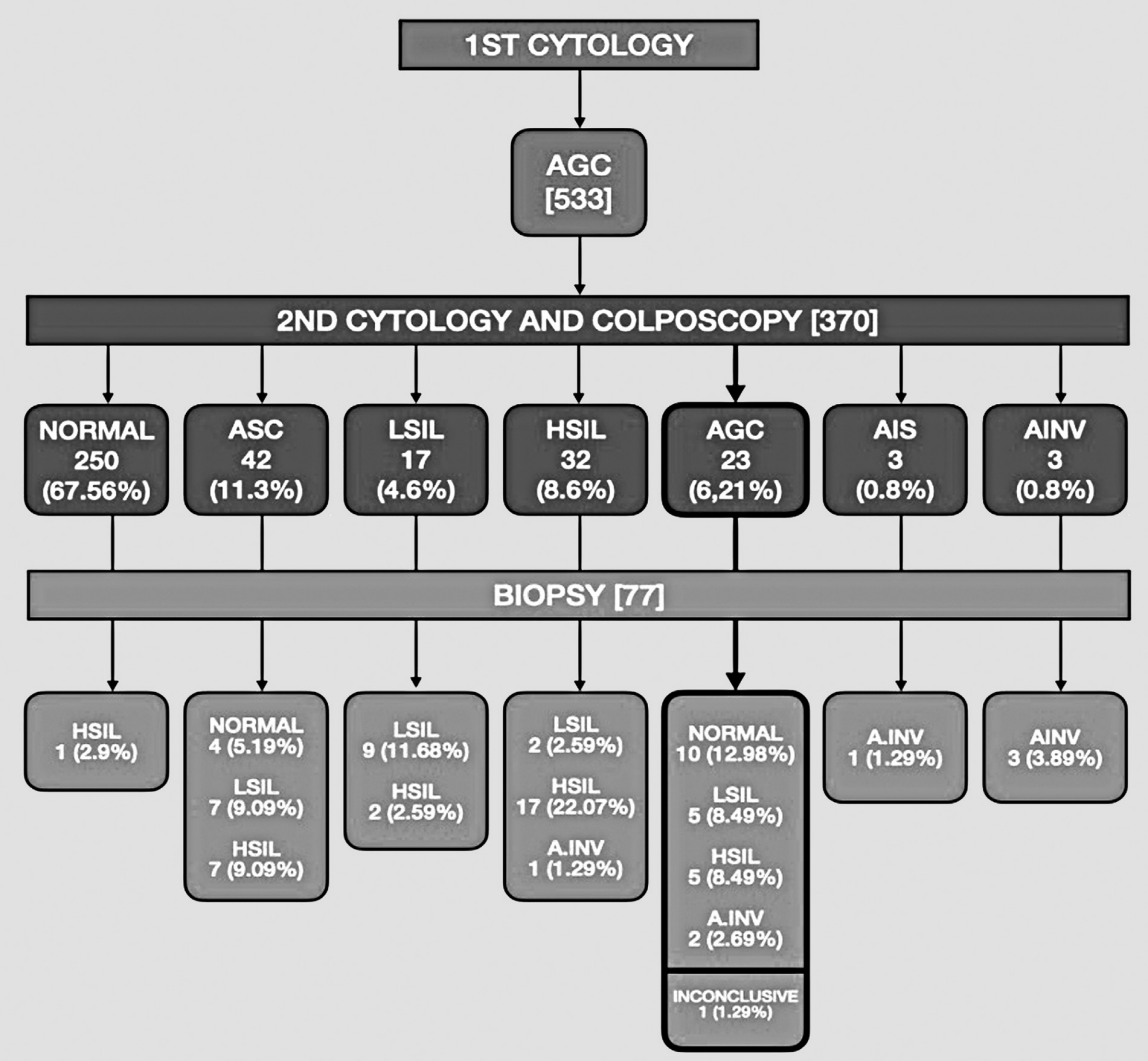
Second cytologic results in 370 women with a previous atypical glandular cell report, followed by histologic results of 77 cases that required biopsy.

A total of 132,147 cytopathological exams were collected and analyzed. Of these, 533 had AGC results. The prevalence of AGC cytology in the studied population was 0.4%. The average age of women with AGC was 40.7 years (range from 14 to 95 years). A total of 69.4% (370/533) women were submitted to a 2 ^nd^ cytologic exam and colposcopy. After to the 2 ^nd^ cytology exam and colposcopy, the following results were obtained: 67.6% (250/370) of normality, 24.5% (91/370) of atypia in squamous cells, 6.2% (23/370) of AGC. 1.6% (6/370) of the patients had a suspected adenocarcinoma (3 in situ, 3 invasive). 30.6% (163/533) of the women did not attend colposcopy/second cytology and were lost to follow-up. A total of 20.8% (77/370) of the women presented colposcopy changes and underwent biopsy and histological studies. Of these, 71.4% (55/77) demonstrated squamous cervical intraepithelial lesion, 9.1% (7/77) invasive adenocarcinoma, 18.2% (14/77) were negative to intraepithelial or invasive lesion, and 4.3% (1/77) had inconclusive results. Of the 23 women with a 2 ^nd^ AGC cytology, 43.4% (10/23) had normal histology, 43.4% (10/23) had a squamous lesion (LSIL or HSIL), 8.7% (2/23) received the diagnosis of invasive adenocarcinoma (INV A), and in 4.3% (1/23) the histological report was inconclusive. The results of the second cytologic examination and biopsy are exposed in Fig. 1 .

Regarding age ranges, 79.22% (417/533) of them were between 25 and 54 years old. Likewise, 82.61% (19/23) of the women who retested positive for AGC were in the same age group. All the women with HSILs or invasive adenocarcinoma confirmed on biopsy also were between 25 and 54 years old. Lastly, among the 49 patients younger than 25 years with a 1 ^st^ AGC result, only 1 retested positive for it with LSIL histology. All the associations regarding age range and test results are exposed in Tables 1 and 2 .

**Table 1:** Distribution of patients with atypical glandular cell results according to age, for the first and second cytologic tests

AGE	1st cytology (AGC) n (%)	2nd cytology (AGC) n (%)
14–24	49 (9.2)	1 (4.3)
25–34	125 (24.4)	5 (21.7)
35–44	152 (28.5)	6 (26.1)
45–54	140 (26.3)	8 (34.7)
55–64	36 (6.7)	0 (0)
> 64	31 (5.8)	3 (13.0)
Total	533 (100)	23 (100)

**Table 2:** Distribution of histologic results according to age range, among women submitted to biopsy after a second cytologic test indicative of atypical glandular cells

Age	Second cytology (AGC)	Histology				
		Normal n (%)	LSIL n (%)	HSIL n (%)	INVA n (%)	Inconclusive n (%)
14–24	1	0	1(4.3)	0	0	0
25–34	5	2 (8.7)	0	1(4.3)	1(4.3)	1(4.3)
35–44	6	2 (8.7)	3 (13.0)	1(4.3)	0	0
45–54	8	3 (13.0)	1 (4.3)	3 (13.0)	1(4.3)	0
55–64	0	0	0	0	0	0
> 64	3	3 (13.0)	0	0	0	0
TOTAL	23	10 (43.5)	5 (21.7)	5 (21.7)	2 (8.7)	1(4.3)

Abbreviations: INVA, invasive; HSIL, high-grade squamous intraepithelial lesion; LSIL, low-grade squamous intraepithelial lesion.

## Discussion

There are not many publications in the literature investigating the clinical significance of a cytopathological diagnosis of AGC. Perhaps this is due to the low frequency in which this finding occurs. Besides, the interpretation of the cytology exam holds a low inter-observer agreement rate, leading to difficulty in reporting AGC. 18

The AGC cytology is a diagnostic challenge due to several reasons: (1) the large variability in cytological criteria; (2) the poverty or absence of colposcopic imaging, inhibiting the teaching and learning of its interpretation; and (3) the array of histological findings that AGC may relate with, from benign diseases to squamous or glandular invasive lesions. 19

Studies that have tried to demonstrate the clinical-histological implications of AGC had mixed outcomes. 19 20 Zhao et al. (2009) 20 showed that despite the low frequency of AGC (0.8%), its clinical importance lies in its high-risk relation to invasive endometrial lesions. These authors showed that the majority of AGC patients who had cancer on biopsy had severe lesions of endometrial origin. However, in the same year, they published a new study demonstrating that even though women with AGC cytology more commonly present with an endometrial disease, this is most likely related to the patient's age (> 50 years) than to AGC itself. 21 In our study, none of the women first diagnosed with AGC had an endometrial disease. Similar to our findings, the study by Zhao et al. 20 found a low prevalence of AGC (0.4%).

Lai et al. (2007) 22 performed a 4-year study, which included 103,073 cytologic studies, 0.1% of which were AGC results. In more than 50% of the cases, the histological diagnosis was negative for intraepithelial or invasive lesions, matching our results (43.47%). Our research, similar to Lai et al. 22 study, showed that AGC can correlate with both squamous and glandular diseases on histology.

Norman et al. (2017) 23 performed a cross-sectional study evaluating the prevalence of AGC in cytologic exams collected in Sweden. They showed a higher association between AGC and normal (46.3%) or HSIL (25.4%) biopsies. Cases of HSIL were only seen in women older than 40 years. This study agrees with those results, as the majority (43.5%) of our AGC patients were biopsy-proven disease-free, and the ones with HSIL or invasive carcinoma were never younger than 25 years.

At this point, there seems to be no consensus regarding the management and outcomes of an AGC finding on screening cytologic examinations. However, since a part of the patients presenting with it may have advanced diseases, it must be considered the active investigation of this diagnosis (by repeating the exam with sampling the endocervical canal and colposcopy) of utmost importance. Nevertheless, our experience demonstrates that this approach is probably most beneficial for those patients older than 25 years. This is because: (1) the majority of younger women will not repeatedly test positive for AGC; (2) they will only rarely have any histologic alteration; if present, (3) it will most likely be of low-grade; and, in this study, we found HSIL in women under 40 years.

In our study, we observed a great number of normal cytology when these were repeated. This was probably due to misinterpretation in cases of AGC in the first cytology. On the other hand, invasive lesions were not present in the older women. This probably due to the age of the population that go to health service for screening in the studied region.

The Brazilian Guidelines for Cervical Cancer Screening (2016) 15 recommend that screening should be performed in women between 25 and 64 years old. Despite this, several younger women were tested in the studied population. Among them, only one had persistency of AGC on the second cytologic test, and its biopsy resulted in an LSIL. Therefore, we question if these younger patients should also be actively followed-up after their first AGC result. In this study, the discomfort of a colposcopy, the risks of a biopsy, and the costs of all the procedures involved seem to weight against the highly unlikely chance of detecting cancer.

In conclusion, the finding of AGC cytology in the uterine cervix is rare. It will most commonly be found in women of reproductive age, between 25 and 54 years old. Most women with AGC will not have correlating alterations on biopsy. Nevertheless, they should be actively investigated (colposcopy, directed biopsy, endocervical cytology), particularly if they are 25 years old or older, due to the important, albeit rare, malignant diseases that they might present with.
